# Using a psychosocial subgroup assignment to predict sickness absence in a working population with neck and back pain

**DOI:** 10.1186/1471-2474-12-81

**Published:** 2011-04-26

**Authors:** Cecilia Bergström, Jan Hagberg, Lennart Bodin, Irene Jensen, Gunnar Bergström

**Affiliations:** 1 Karolinska Institutet, Division of Intervention and Implementation Research, Department of Public Health, SE - 171 77 Stockholm, Sweden

## Abstract

**Background:**

The overall objective was to evaluate the predictive validity of a subgroup classification based on the Swedish version of the MPI, the MPI-S, among gainfully employed workers with neck pain (NP) and/or low back pain (LBP) during a follow-up period of 18 and 36 months.

**Methods:**

This is a prospective cohort study that is part of a larger longitudinal multi-centre study entitled Work and Health in the Process and Engineering Industries (AHA). The attempt was to classify individuals at risk for developing chronic disabling NP and LBP. This is the first study using the MPI-questionnaire in a working population with NP and LBP.

**Results:**

Dysfunctional individuals (DYS) demonstrated more statistically significant sickness absence compared to adaptive copers (AC) after 36 months. DYS also had a threefold increase in the risk ratio of long-term sickness absence at 18 months. Interpersonally distressed (ID) subgroup showed overall more sickness absence compared to the AC subgroup at the 36-month follow-up and had a twofold increase in the risk ratio of long-term sickness absence at 18 months. There was a significant difference in bodily pain, mental and physical health for ID and DYS subgroups compared to the AC group at both follow-ups.

**Conclusions:**

The present study shows that this multidimensional approach to the classification of individuals based on psychological and psychosocial characteristics can distinguish different groups in gainfully employed working population with NP/LBP. The results in this study confirm the predictive validity of the MPI-S subgroup classification system.

## Background

Neck and low back pain is a common cause of long term sickness absence as well as exclusion from the labor market, both in Sweden and internationally [1]. In Sweden alone, the cost of neck and low back pain has been estimated to 1.3% of GNP [2]. Thus, the prevention of chronicity has become important, in order to reduce costs and to lessen the suffering for individuals with neck pain (NP) and low back pain (LBP) [3]. It has been suggested that early preventive interventions may reduce future problems as well as selection criteria are of outmost importance for the outcome [4].

Psychological factors have long been associated with chronic pain and they also seem to exacerbate the clinical component of pain [5,6]. In fact, psychosocial factors have shown not only to be pivotal in the transition from acute and subacute NP and LBP to chronicity but also have a strong influence on the onset of pain [5,7,8]. Furthermore, coping or elements of coping have been shown to be a strong to moderate predictor for future LBP [9,10].

Attempts have been made to classify patients into subgroups to better understand which subtypes of patients would benefit from what particular treatment [11-13]. The Multidimensional Pain Inventory (MPI) [14] was originally developed for chronic pain patients and is widely used to derive subgroups of patients [15]. Three different subgroups derived empirically from the MPI have been labeled: dysfunctional (DYS), interpersonally distressed (ID) and adaptive copers (AC) [15]. The DYS subgroup are characterized by high pain severity, disability and affective distress, and ID individuals are characterized by low levels of social support, while the AC subgroup report a more successful adjustment to chronic pain.

The overall objective of the study was to evaluate the predictive validity of a subgroup classification based on the Swedish version of the MPI (the MPI-S) [16,17] among gainfully employed workers with NP and LBP during a follow-up period of 18 and 36-month.

Due to poorer coping ability and higher pain severity in both ID and DYS individuals, compared to AC individuals, it is hypothesized that DYS and ID subgroups should have more sickness absence than the AC subgroup. Secondly, it was hypothesized that DYS and ID individuals should score worse in regard to bodily pain, mental and physical health compared to the AC subgroup at the 18 and 36-month follow-ups.

To the best of the authors' knowledge, this is the first study that uses the Swedish version of the MPI (MPI-S) on a gainfully employed working population.

## Method

This prospective cohort study is part of a larger longitudinal multi-centre study entitled the AHA-study (Swedish abbreviation for Work and Health in the Processing and Engineering Industries [18]. It was carried out at four large workplaces in Sweden during the years 2000 to 2003 and included over 4000 employees. Two companies were paper mill industries, one was a steelwork company and one was a truck manufacturer.

### Procedure

The participants answered a survey addressing health, lifestyle and work-related factors which was sent out by mail to the entire workforce. This screening instrument has been described elsewhere [18] and was a compilation of valid questionnaires, measuring health related variables, lifestyle, and work characteristics. It also included the identification of subjects with a potential risk for developing illness and sickness absence. The survey was administered three times and data was collected at baseline, after 18-months and at 36-months follow-up within the AHA study.

### Subjects

Of the 2894 responders at baseline, 273 (9.4%) were considered at risk for developing chronic disabling NP and/or LBP, and sick leave. This risk was measured by questions specifically related to the prevalence and the degree of complaint as well as to what extent it had led to sickness absence due to NP/LBP either presently or during the previous year [18].

### Measurement instruments

#### The Swedish version of the MPI (MPI-S)

The development of the MPI-S has been described earlier [16,17]. The reliability and validity of the MPI-S has been shown to be acceptable. It comprises 34 items and includes one psychosocial and one behavioral section. Part 1 consists of five scales: pain severity (PS), pain-related interference in everyday life (I), perceived life control (LC), affective distress (AD), and perceived support from significant others (S). Part 2 encompasses three scales that measure the patient's perception of responses of significant others to displays of pain and suffering. The three scales are punishing responses (PR), solicitous responses (SR) and distracting responses (DR). All scales contained an interval of 0 to 6, where a high score indicates more of the characteristics in question.

#### Sick leave

Information about sick leave was gathered from the companies' payrolls. Thus, all sickness absence is on record except for those whose employment was terminated during the study period.

#### Short form-12 (SF-12)

Bodily pain, physical and mental health were estimated by using scales from the Short Form-12 (SF-12) [19]. SF-12 was developed as a shorter alternative to the SF-36. It can be administered in 2 minutes or less, thus making it easy to use in large-scale studies [19,20]. The scale is scored positively so that a higher score indicates better physical and mental functioning and less bodily pain.

### Content of the Clinical Investigation

The investigation of individuals with NP/LBP complaints referred to the OHS consisted of one medical investigation focusing on indications of specific diseases, so-called "red flags" [21,22] and one psychosocial investigation focusing on risk factors for chronicity, so called "yellow flags" [5,6]. All of the examined employees were advised to stay active and were given a "back book" that offered evidence-based advice on coping with back pain and leading a normal life. Participants who declined further contact with the OHS were also offered a "back-book". In this study, 17 individuals participated in a multidisciplinary rehabilitation (ID = 5, DYS = 3, and AC = 9).

### Statistical methods

#### MPI-S classification system

Classifications of the participants were done using a software program employing discriminant analysis based on the participants profile found from the clusters analysis as reported by Bergström et al [23].

Due to violation of normality assumption, all statistical analysis regarding sickness absence, mental and physical health as well as bodily pain (at both 18-month and 36-month follow-up) were all done by Kruskal-Wallis 1-way ANOVA on ranks, as this non-parametric test is a suitable alternative for comparisons of three or more groups [24]. For similar reasons the Mann-Whitney *U *test applied with the Holm-Bonferroni correction for multiple tests was chosen for group-wise posthoc analyses. The Holm-Bonferroni allows more rejection of the null hypothesis, and thus is less conservative and more powerful than the Bonferroni method [25]. In order not to reject the null hypothesis too easily, a double-sided test was used instead of a one-sided test proposed by the hypothesis in the introduction. The AC subgroup was used as a predefined reference group in all analyses as individuals belonging to this group were considered better copers. A modified Poisson regression [26] was performed to predict the risk ratio for long-term sickness absence during the follow-up period. Long-term sickness absence was defined as >30 days, thus the outcome variable was dichotomized into ≤30 days and >30 days of sickness absence [27-31]. The final model of the modified Poisson regression analysis the dichotomization of sickness absence was supported by figures based on goodness of fit, where the 30-day cut-off had the lowest AIC and BIC values [32]. Earlier studies have shown that individuals with less than 30 days of LBP are candidates for short-term recovery [33]. Furthermore, sickness absence for >30 days is commonly used as an outcome variable for long-term sickness absence [28,30]. In all of the analyses, the PASW 17.0 software package was used. Statistical significance was set at *p *< 0.05 when comparing differences between two groups and/or among the three MPI-S groups.

### Ethical approval and informed consent

Approval of all ethical considerations regarding this study was obtained from the Committee on Ethics at Karolinska Institutet, Stockholm (AHA; Dnr 00-012). A written consent was obtained from all participants.

## Results

### Classification of participants

**Figure 1:**
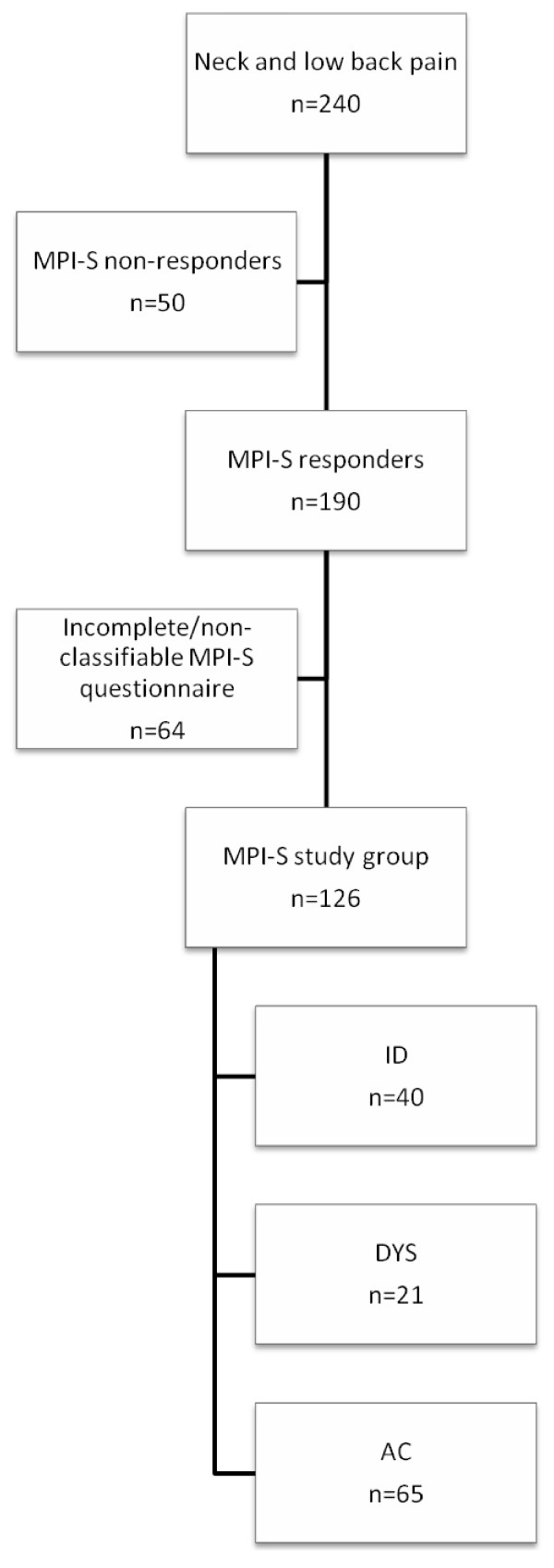
**Selection chart of the study group**.

In total, 240 of the 273 at-risk individuals were able to be contacted by the research team and were mailed the MPI-S questionnaire as well as offered further investigation at the Occupational Health Services (OHS). A total of 190 (79%) questionnaires were returned, of which 41 were excluded due to missing data on section 2 of the MPI-S. Furthermore, 23 subjects were classified as Hybrid (response pattern represents aspects from more than one of the three MPI-S profiles) were excluded, as it has been argued that they do not render any valid clinical information [34]. Consequently, 126 individuals constituted the study group. Neither the treatment staff, nor participants, nor anyone in the research team was informed of the participants' subgroup affiliation during the investigation and data collection (Figure 1).

Table 1 describes the MPI-S study population at baseline, where 40 individuals were classified as interpersonally distressed (ID), 21 as dysfunctional (DYS), and 65 individuals as adaptive copers (AC). The study population was male-dominated and the mean age across the MPI-S subgroups was 41.5 years (SD 9.4). The majority of the employees were blue-collar workers, over 90% were married or cohabiting, and close to 60% had at least a high school education. Individuals with incomplete questionnaires or with a Hybrid profile were male-dominated (81.3%) with a mean age of 41.2 (SD 9.9), the majority were blue-collar workers, 51.6% were married or cohabiting with or without children, and 60.3% had at least a high school education. The majority had experienced NP/LPB the past year and 95.3% had been sick absent the past year due to NP/LBP.

**Table 1:** Descriptive information on MPI-S subgroups at baseline

	MPI-S study group			
	ID	DYS	AC	Total
	*n *= 40	*n *= 21	*n *= 65	*n *= 126
**Gender**				
Men	35 (87.5)	15 (71.4)	62 (95.4)	112 (88.9)
**Age**, mean (SD)	40.9 (9.6)	45.7 (9.3)	40.6 (9.1)	41.5 (9.4)
**Marital status**				
Single, living alone or with children	6 (15)	2 (9.5)	3 (4.6)	11 (8.7)
Co-habiting with other adult	10 (25)	5 (23.8)	21 (32.3)	36 (28.6)
Co-habiting with other adult and children	24 (60)	14 (66.7)	41 (63.1)	79 (62.7)
**Education**				
Compulsory school	14 (35.0)	8 (40.0)	26 (40.0)	48 (38.1)
High school	24 (60.0)	12 (60.0)	35 (53.8)	71 (56.3)
Post high school	2 (5.0)	-	4 (6.2)	6 (4.8)
**Employment grade**				
Blue collar	38 (95.0)	20 (95.2)	60 (92.3)	118 (93.7)
White collar	2 (5.0)	1 (4.8)	5 (7.7)	8 (6.3)
**Heavy lifting **(>10 kg)				
Almost never	16 (40)	7 (33.3)	21 (32.3)	44 (35.2)
1-5 times/day	13 (32.5)	6 (28.6)	24 (36.9)	43 (34.4)
6-10 times/day	2 (5)	1 (4.8)	5 (7.7)	8 (6.4)
>10 times/day	8 (20)	7 (33.3)	15 (23.1)	30 (24)
**Working with arms above shoulders**				
Almost never	18 (45)	3 (14.3)	31 (48.4)	52 (41.3)
5-30 min/day	15 (37.5)	20 (30.8)	20 (31.3)	41 (32.5)
31-60 min/day	2 (5)	5 (23.8)	4 (6.2)	11 (8.7)
>60 min/day	5 (12.5)	7 (33.3)	9 (14.1)	21 (16.7)
**Smoking**				
Yes	3 (7.5)	6 (30)	10 (15.9)	19 (15.5)
No, stopped less than 12 months ago	1 (2.5)	-	-	1 (0.8)
No, stopped more than 12 months ago	15 (37.5)	10 (50)	17 (27)	42 (34.1)
No, never been a smoker	21 (52.5)	4 (20)	36 (57.1)	61 (49.6)

Numbers in parenthesis are percentage unless otherwise specified.

Table 2 shows gives an overview of the amount of neck and back pain experienced in each of the MPI-S subgroups, as well as amount of sickness absence due to neck and back pain. The majority of individuals had mixed pain sites (66%) and had experienced LBP and NP once or several times the past year (94% and 72% respectively). In addition, the vast majority had been sick absent due to NP/LBP (93%). It also reveals that DYS individuals have statistically significant more NP compared to AC individuals.

**Table 2:** Descriptive information on MPI-S subgroups

	MPI-S subgroups				
	ID	DYS	AC		
	
	*n *= 40	*n *= 21	*n *= 65	**χ**^**2 **^**values**	*p*-value
**Back pain the past year**, *n *(%)				2.68	0.262
No	2 (5.0)	3 (14.3)	3 (4.6)		
Yes, once or several times	38 (95.0)	18 (85.7)	62 (95.4)		
**Neck pain the past year**, *n *(%)				**6.75**	0.034*
No	9 (22.5)	2 (9.5)	24 (36.9)		
Yes, once or several times	31 (77.5)	19 (90.5)	41 (63.1)		
**Mixed pain site the past year**, *n *(%)				3.37	0.186
No	11 (27.5)	5 (23.8)	27 (41.5)		
Yes, once or several times	29 (72.5)	16 (76.2)	38 (58.5)		
**Sickness absence due to neck/back pain**, *n *(%)				4.55	0.103
No	-	2 (9.5)	7 (10.8)		
Yes, once or several times	40 (100)	19 (90.5)	58 (89.2)		

The MPI-S software was used in the classification of the patient group to obtain the subgroups ID, DYS, and AC.

* Significance test *p *< 0.05

### Attrition

Information on the primary outcome, sickness absence, was available for 113 individuals during the period 0-18 months follow-up and 109 individuals 19-36 month follow-up. A total of 17 individuals stopped their employment during the follow-up period (ID = 4, DYS = 5, AC = 8). The response rate on the SF-12 at 18 months was 76.2%, and 73% at 36 months. Non-respondents at 18 months consisted of 11 ID individuals, 7 DYS individuals, and 12 AC individuals. At 36 months, 12 belonged to the ID subgroup, 8 to the DYS subgroup, and 14 belonged to the AC subgroup. The mean age at baseline for the 18 months non-respondents was 40.1 (SD 11.1), and at 36 months 44.2 (SD 10.7).

### Sickness absence

**Figure 2:**
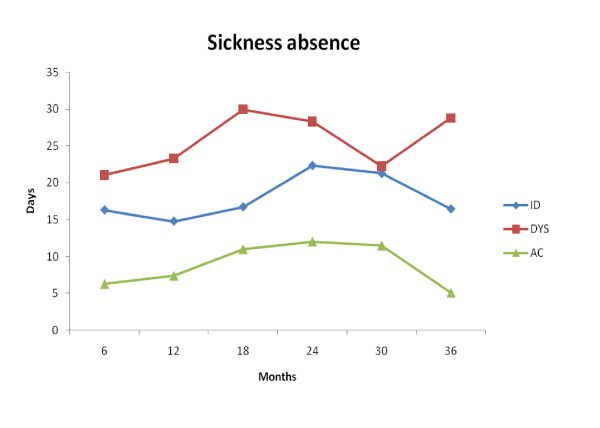
**Sickness absence in days for every 6 months for each of the MPI-S groups**.

Figure 2 gives a visual overview of sickness absence during the 36-month follow-up. The AC group has less sickness absence compared to the other two MPI-S groups and it is evident that the MPI-S subgroups follow a distinctive course of sickness absence during the follow-up period. A visual inspection suggests that the ID and DYS individuals had more sickness absence compared to the AC group during the entire follow-up period.

A Kruskal-Wallis one-way ANOVA rank test was performed on the results of the MPI-S subgroups (Table 3). The analysis approaches a statistically significant effect on the overall sickness absence at 18 months (*p *= 0.056). However, when performing Mann-Whitney *U *test between groups, there was a significant statistical difference between DYS and AC with median and Interquartile Range (IR) in parenthesis: (40.22 (IR = 113.01), *p *= 0.04). Conversely, this was not supported by the Holm-Bonferroni method.

**Table 3:** Comparing number of days on sick leave, mental and physical health, and bodily pain using adaptive copers (AC) as a reference group

	MPI-S subgroups										
	ID					DYS					
			
	n	**Median**^**a**^	**IR**^**b**^	*p*-value MW-test	Holm-Bonferroni	n	**Median**^**a**^	**IR**^**b**^	*p*-value MW-test	Holm-Bonferroni	***p*-value **^**c **^**KW-test**
**Sickness absence**											
0-18 months	36	23.44	32.29	0.083	Not rejected	18	40.22	113.01	**0.04**	Not rejected	0.056
19-36 months	36	22.56	60.78	**0.024**	Rejected	16	30.23	125.54	**0.014**	Rejected	**0.013**
											
**Mental Health**											
18 months	29	62.50	29.38	**0.007**	Rejected	14	43.75	35.94	**0.001**	Rejected	**<0.001**
36 months	28	66.88	38.13	**<0.001**	Rejected	13	41.25	40.63	**0.002**	Rejected	**<0.001**
											
**Physcial Health**											
18 months	29	52.50	27.50	**0.007**	Rejected	14	28.13	22.50	**<0.001**	Rejected	**<0.001**
36 months	28	59.38	44.06	**0.020**	Rejected	13	25.00	40.63	**0.001**	Rejected	**0.001**
											
**Bodily Pain**											
18 months	29	50.00	37.50	**0.004**	Rejected	14	37.50	50.00	**<0.001**	Rejected	**<0.001**
36 months	28	50.00	43.75	**0.007**	Rejected	13	25.00	37.50	**<0.001**	Rejected	**<0.001**

Interpersonally distressed (ID) and Dysfunctional (DYS).

^a ^Median Mann-Whitney *U *test. ^b ^IR = Interquartile Range. ^c ^Kruskal-Wallis *p*-value.

Significant result *p *< 0.05 using Mann-Whitney *U *test.

Significant result *p *< 0.05 using Kruskal-Wallis test.

The same analyses was performed of the overall sickness absence at 36 months and revealed statistically significant difference between the MPI-S groups (*p *= 0.013) as well as between ID and AC (median 22.56 (IR = 60.78), *p *= 0.024) and DYS and AC (median 30.32 (IR = 125.54), *p *= 0.014). These findings were supported by the Holm-Bonferroni analysis.

Modified Poisson regression analysis was performed with days of sickness absence at 18 months and 36 months as the dependent variable and MPI-S subgroups as predictor variables, after dichotomizing individuals who had been absent for sickness for ≤30 days and for >30 days (Table 4). All individuals were analyzed and the full model for the 18-month follow-up significantly predicted future sickness absence (in days) for all MPI-S subgroups when compared to AC patients, *p *= 0.002. The risk ratio (RR) for sickness absence at 18 months increased for the ID and DYS patient groups in comparison to the AC patient group 95% confidence interval (CI) in parenthesis; RR = 2.086, *p *= 0.032 (CI: 1.065 to 4.085) for ID, and RR = 3.278, *p *< 0.001 (CI: 1.715 to 6.266) for DYS. The model for 36-month follow-up did not significantly predict future sickness absence. However, there was an increased risk ratio for the DYS patient group in comparison to the AC group at the 36-month follow-up, CI in parenthesis; RR = 1.900, *p *= 0.055 (CI: 0.987 to 3.657), though, this was not statistically significant. We also tried a multiple model including rehabilitation and sickness absence prior to baseline as explanatory variables. However, this did not change the above statistically significant results regarding the risk ratio of future sickness absence.

**Table 4:** Risk ratios for sickness absence of more than 30 days for the MPI-S subgroups, estimated by modified Poisson regression

	ID		DYS	
		
	RR	CI (95%)	RR	CI (95%)
				
0-18 months	2.086*	1.065 - 4.085	3.278*	1.715 - 6.266
19-36 months	1.689	0.957 - 2.979	1.900	0.987 - 3.657

*Significant at the 5% level, where more than 30 days of sickness absence is outcome and 30 days or less is the reference category.

AC is the reference of the explanatory variable.

### Mental and physical health (SF-12)

**Figure 3:**
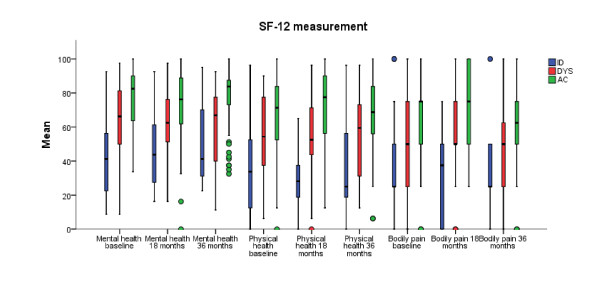
**Bodily pain, mental and physical health measured by SF-12 for each of the MPI-S groups**.

Figure 3 gives a visual overview of the two different SF-12 scales mental and physical health scales, demonstrating that the ID and DYS group scored lower compared to the AC group. Statistically significant results were found between the MPI-S groups in regard to both mental and physical health at 18 and 36-months follow-up (Table 3). Further, statistically significant results were found between ID and AC (Mental Health: 18 months; median: 62.50 (IR = 29.38), *p *= 0.007 and 36 months; median: 66.88 (IR = 38.13), *p *< 0.001, Physical Health: 18 months; median: 52.50 (IR = 27.50), *p *= 0.007 and 36 months; median 59.38 (IR = 44.06), *p *= 0.020), and DYS and AC (Mental Health: 18 months; median: 43.75 (IR = 35.94), *p *= 0.001 and 36 months; median: 41.25 (IR = 40.63), *p *= 0.002, Physical Health: 18 months; median: 28.13 (IR = 22.50), *p *< 0.001 and 36 months; median 25.00 (IR = 40.63), *p *= 0.001) (Table 3).

### Bodily pain

Bodily pain was measured by the SF-12 and Figure 3 illustrates that the AC group scored better with regard to bodily pain in comparison to both ID and DYS patient groups at 18 and 36-month follow-ups. Further analyses rendered a statistically significant difference between the MPI-S groups (*p *< 0.001 at both 18 and 36-months follow-up). Statistically significant results were found between ID and AC (18 months; median: 50.00 (IR = 37.50), *p *= 0.004 and 36 months; median: 50.00 (IR = 43.75), *p *= 0.007) and DYS and AC (18 months; median: 37.50 (IR = 50.00), *p *< 0.001 and 36 months; median: 25.00 (IR = 37.50), *p *< 0.001) (Table 3).

## Discussion

The aim of this study was to evaluate the predictive validity of the MPI-S with regard to sickness absence, bodily pain, and mental and physical health on a gainfully employed working population with NP and/or LBP at 18 and 36-month follow-ups. As hypothesized, individuals with more pronounced psychosocial difficulties (DYS) demonstrated statistically significant more sickness absence compared to AC patients at 36 months. DYS patients also had a threefold increase in the risk ratio of long-term sickness absence (>30 days) at 18 months follow-up. Individuals belonging to the ID group showed overall more sickness absence compared to the AC patients at the 36-month follow-up. Furthermore, the ID subgroup had a twofold increase in the risk ratio of long-term sickness absence at 18 months. Figure 2 gives a visual overview of the trend regarding sickness absence throughout the study period for all of the MPI-S subgroups. It is noteworthy that the AC subgroup had consistently less sickness absence compared to the other MPI-S groups.

The secondary hypothesis was also confirmed demonstrating a significant difference in mental and physical health as well as bodily pain for ID and DYS individuals compared to the AC subgroup at both 18 and 36-month follow-ups. This further supports the definition of these three MPI-S groups in a working population.

In comparison with studies analyzing individuals with chronic pain using the MPI-S questionnaire [35], the proportion of participants in the current study's AC subgroup is high. This seems reasonable, as this population probably includes a large proportion of individuals who may not have developed inappropriate coping strategies or pain behavior. In other studies, the AC subgroup report a high level of social support, and relatively low levels of pain. They also seem to remain active despite pain [36,37]. Further, it is evident that AC individuals in this study had consistently less sickness absence compared to both DYS and ID individual. This might possibly be due to important determinants for claiming sick leave due to LBP [38], i.e. AC individuals are characterized by better coping abilities, a more positive outlook in regard to LBP, less pain and co-morbidities.

LBP is not a self-limiting condition [39-41] as a large proportion (42-75%) still experience pain after 12 months and a majority (44-78%) experiences relapses of pain [39]. Further, recurrence of LBP is strongly correlated with previous episodes of LBP [39,42]. The population in this study consisted of gainfully employed workers with NP/LBP, and the majority of the individuals could be considered non-chronic in nature. Nevertheless, due to previous episodes many individuals with recurrent NP and LBP may have similar experiences of pain as chronic patients, thus making the MPI-S questionnaire a useful tool in this kind of population as well.

ID and DYS individuals may appear similar when comparing pain, disability and emotional distress, but their coping ability seems to differ, i.e. DYS patients often rely more on fear-avoidance coping strategies compared to both ID and AC patients [37,43]. Further, DYS patients have been found to be significantly more depressed compared to ID patients and are more likely to dwell on somatic symptoms or suffer from hypochondriasis [37]. However, there may be different factors associated with depression in these two groups [23], i.e. depression for ID patients could possibly more connected to marital and interpersonally difficulties compared to DYS patients. In addition, ID patients reported a lack of support from their significant other and rated their interpersonal relationships to be of lower quality compared to both DYS and AC patients [37]. However, the distinction between ID and DYS patient groups have recently been questioned in a recent study by Junhaenel et al [36]. The study found no statistically significant difference in some measures regarding interpersonal relationship between the two groups [36]. However, the sample size in the cited study was small which could have contributed to the non-significant results. Nevertheless, predictive results from previous studies have shown that ID and DYS patients do differ in the development of future sickness absence [23,35].

Recurrent and persisting symptoms of NP and LBP may be more related to psychosocial factors than medical aspects [21,44]. Thus, when trying to predict the likelihood of progression from acute/subacute NP/LBP into chronicity and in turn future long-term sickness absence, it is necessary to address psychosocial factors. Heitz et al suggests that a psychosocial intervention may be more effective in a subacute phase but that an interdisciplinary approach is justified in both subacute and chronic LBP stages [45]. Thus, DYS and ID considered at-risk may be offered early vocational rehabilitation, counseling when needed, interventions related to improve interpersonal relationships for ID patients, and scheduled for a follow-up visit with the OHS. Collaboration with other caregivers outside the OHS could also be established to enable early and more customized intervention, hence reducing the risk of long-term sickness absence.

The present study shows that this multidimensional approach to the classification of individuals based on psychological and psychosocial characteristics can distinguish different groups in gainfully employed working population with NP/LBP. Furthermore, the MPI-S should be viewed as a tool to classify patients into valid subgroups matching treatment plans to subgroup characteristics. The logical corollary would be that early, customized interventions for patients with NP and LBP would improve health outcomes, which is an important objective for any individual as well as healthcare system. Hence, by using the MPI-S classification system together with other clinical data, customized treatments may enable patients to break negative patterns of pain coping strategies, and thus reduce future long-term sickness absence.

There are some methodological considerations in this study that should be acknowledged. Firstly, the sample is male-dominated and the vast majority of subjects were blue-collar workers in the Process and Engineering Industries in Sweden. Thus, this could decrease the generalizability of the results on a more evenly distributed population with regard to gender and among other working populations, e.g. health care and service sector. Furthermore, the reason for sickness absence was not known as this information was not provided from the companies' pay-roll. Consequently, data on sickness absence in this study may also mirror other health complaints among these employees. Non-responsiveness in regard to mental and physical health as well as bodily pain at 18 and 36-month follow-ups may have introduced some bias. However, the non-response at 18 and 36-month follow-ups was proportionally similar between the MPI-S groups. Nevertheless, the results show that the DYS and ID groups display higher sickness absenteeism than the AC group during follow-up.

The data material did not contain information in regard to severity and duration of the individuals back and neck complaint which are in and by itself a limitation. If information on severity, chronicity of neck and back complaints in combination with vocational and comorbid factors would have been available it would have enhanced the internal validity of the study. However, previous studies have confirmed the internal reliability, validity and generalizability of the MPI-S instrument in a chronic population [23]. Furthermore, during the validation process of the MPI-S, the subgroups did not differ on pain duration or medical variables [23].

Failure by an individual to respond to section two of the MPI questionnaire leads to an unclassifiable profile [46,47]. In this study, 41 individuals were excluded who could not be classified at the first assessment due to missing data on section two of the MPI-S, which requires a significant other. In addition, another 23 individuals classified as Hybrid (response pattern represents aspects from more than one of the three MPI-S profiles) were also excluded, as it has been argued that they do not render any valid clinical information [34]. Unfortunately, this contributed to small MPI-S subgroups which reduced the statistical power and increased the risk for Type II error, hence increasing the risk of failing to reject the null hypothesis. This exclusion of individuals could probably have been reduced, if the refinement of instructions in section two of the MPI, as described by Okifuji et al [47], had been used in this study. A general consideration is that it has been shown that approximately one third of patients classified to one of the three MPI patient groups change within a month [46] and that the majority of patients who change classification belong to the Hybrid category [47]. This could potentially have been detected by administrating the MPI-S questionnaire within this time period, hence further increasing the statistical power.

## Conclusions

In conclusion, to the best of the authors' knowledge, this is the first study using the MPI-questionnaire on gainfully employed working population with NP and LBP. The results in this study confirmed the predictive validity of the MPI-S subgroup classification system among gainfully employed workers with NP and LBP during a follow-up period of 18 and 36 months.

## Competing interests

The authors declare that they have no competing interests.

## Authors' contributions

All authors have read and approved the final manuscript. CB was involved in analysis and interpretation of the data, drafting and revising manuscript and has given final approval. JH analyzed and interpreted the data, revised manuscript and gave final approval. LB was involved in the interpretation of data and revision of manuscript and gave final approval. IJ was involved in design, data collection, and revision of manuscript and gave final approval. GB was involved in the design, data collection, revision, interpretation of data, and revision of manuscript and gave final approval.

## Pre-publication history

The pre-publication history for this paper can be accessed here:

http://www.biomedcentral.com/1471-2474/12/81/prepub
